# Cancer antigen profiling for malignant pleural mesothelioma immunotherapy: expression and coexpression of mesothelin, cancer antigen 125, and Wilms tumor 1

**DOI:** 10.18632/oncotarget.20845

**Published:** 2017-09-12

**Authors:** Takashi Eguchi, Kyuichi Kadota, Marissa Mayor, Marjorie G. Zauderer, Andreas Rimner, Valerie W. Rusch, William D. Travis, Michel Sadelain, Prasad S. Adusumilli

**Affiliations:** ^1^ Thoracic Service, Department of Surgery, Memorial Sloan Kettering Cancer Center, New York, NY, USA; ^2^ Division of Thoracic Surgery, Department of Surgery, Shinshu University School of Medicine, Matsumoto, Japan; ^3^ Department of Pathology, Memorial Sloan Kettering Cancer Center, New York, NY, USA; ^4^ Department of Diagnostic Pathology, Faculty of Medicine, Kagawa University, Kagawa, Japan; ^5^ Thoracic Oncology Service, Division of Solid Tumor Oncology, Department of Medicine, Memorial Sloan Kettering Cancer Center, New York, NY, USA; ^6^ Department of Radiation Oncology, Memorial Sloan Kettering Cancer Center, New York, NY, USA; ^7^ Center for Cell Engineering, Memorial Sloan Kettering Cancer Center, New York, NY, USA

**Keywords:** mesothelin, CA125, WT1, mesothelioma, chimeric antigen receptor

## Abstract

**Background:**

To develop cancer antigen-targeted immunotherapeutic strategies for malignant pleural mesothelioma (MPM), we investigated the individual and coexpressions of the cancer-associated antigens mesothelin (MSLN), cancer antigen 125 (CA125), and Wilms tumor 1 (WT1) in both epithelioid and non-epithelioid MPM.

**Methods:**

All available hematoxylin and eosin-stained slides from patients who were diagnosed with MPM (1989-2010) were reviewed. We constructed tissue microarrays from 283 patients (epithelioid = 234; non-epithelioid = 49). Intensity and distribution for each antigen were assessed by immunohistochemistry.

**Results:**

Positive expression of MSLN, CA125, and WT1 were demonstrated in 93%, 75%, and 97% of epithelioid MPM cases, and 57%, 33%, and 98% of non-epithelioid MPM cases, respectively. Triple- and double-positive antigen coexpressions were demonstrated in 72% and 23% of epithelioid MPM cases and 29% and 33% of non-epithelioid MPM cases, respectively. Complete absence of expression for all three antigens was demonstrated in <2% of MPM cases. More than two-thirds of MPM cases had ≥50% distribution of MSLN-positive cells and, among the remaining third, half had ≥50% distribution of WT1-positive cells. CA125/MSLN coexpression was observed in more than two-thirds of epithelioid MPM cases and one-third of non-epithelioid MPM cases.

**Conclusion:**

A limited number of cancer-associated antigens can target almost all MPM tumors for immunotherapy.

## INTRODUCTION

Malignant pleural mesothelioma (MPM) is an aggressive malignancy with a median survival of 9 to 12 months [1]. Although multimodality clinical trials for early-stage MPM have shown encouraging results [2–6], its benefits are limited [7, 8]. Additionally, considering that the majority of patients are diagnosed at a late stage, multimodality therapy, including surgical resection, is only an option for select patients [9].

For other thoracic malignancies, such as lung cancer, variable driver mutations have been discovered and corresponding targeted molecular agents have been applied in clinical practice. Recent comprehensive genomic analysis of transcriptomes and exomes from patient MPM samples have identified significantly mutated genes, recurrent mutations, gene fusions, and splicing alterations [10]. However, there is currently no clinically accepted targeted molecular therapy for MPM even though multiple clinical and preclinical studies have attempted to target recently discovered molecular alterations and gene overexpressions [11].

Despite poor prognosis for patients with MPM, tumor-infiltrating immune cells have been investigated and have been shown to be prognostic indicators [12–15]. Recent immunotherapeutic strategies against MPM have demonstrated the immunogenicity of MPM [16] and further suggest that promotion of antigen-specific T-cell responses may prove beneficial. Adoptive T-cell therapy is an immunotherapeutic strategy that includes chimeric antigen receptor (CAR) T-cell therapy [17] and T-cell receptor (TCR) therapy. These therapies utilize genetic engineering to target a patient's own T cells to a tumor-associated antigen. Although results from antigen-targeted cellular immunotherapy studies have shown promise in treating hematologic malignancies [18], candidate target antigens for solid tumors, such as MPM, are limited. Unlike CD19 in leukemia and lymphomas, there is no single antigen present on all tumor cells. Even when an antigen is expressed in the majority of cells, the expression intensity and distribution are not uniform and this may allow antigen escape of low-antigen expressing cells. Strategies that target two cancer-associated antigens have been advantageous [19] and coexpression and distribution of antigens are very important to the further development of dual-antigen targeting strategies. During our search for candidate antigens, we investigated the overexpression of the cell-surface glycoproteins mesothelin (MSLN) and cancer antigen 125 (CA125; also known as mucin-16 [MUC16]), and the transcription factor Wilms tumor 1 (WT1).

MSLN is overexpressed in a broad spectrum of solid tumors including mesothelioma and lung cancer [20–24]. Our rationale for targeting MSLN in MPM is based on our published observations that have shown that: (1) MSLN promotes MPM cell invasion and matrix metalloprotease secretion; (2) MSLN is overexpressed in >90% of patients with epithelioid MPM [23]; and (3) MSLN-targeted CAR T cells delivered regionally to eradicate MSLN-positive pleural tumors align with the regionally aggressive biology of MPM [17]. Supported by this rationale, we are conducting a Phase I clinical trial (NCT02414269) to evaluate the safety of regionally administered MSLN-targeted CAR T cells in patients with either primary pleural malignancies (MPM) or secondary pleural malignancies (lung and breast cancers) with MSLN expression. Other investigators have been conducting MSLN-targeted CAR T-cell therapy clinical trials with systemic administration of CAR T cells (NCT01583686, NCT02159716, NCT01355965, and NCT02930993). In order to expand our immunotherapeutic approaches to treating non-epithelioid, MSLN-negative, and MSLN-focally positive MPM, identifying alternative or additional antigens to target is necessary. Neither overexpression of other cancer-associated antigens nor correlative expressions between those antigens and MSLN have been fully investigated.

CA125 overexpression was initially recognized in ovarian cancer and has recently been reported in several other cancers such as pancreatic [25] and lung cancer [26, 27]. Prior studies have suggested that CA125 may facilitate peritoneal metastasis via binding to MSLN in ovarian cancer [28, 29] and the CA125-MSLN interaction is associated with increased invasion and worse prognosis in pancreatic cancer [30, 31]. Published evidence supports the notion that MSLN- and CA125-specific immune responses are beneficial [32–34] and that promising preclinical and ongoing early-phase clinical studies have demonstrated that MSLN and CA125 may be effective cancer antigens to target with immunotherapy for solid tumors [35–37]; however, the frequency and distribution of CA125 and MSLN co-expression in MPM have not been previously studied.

WT1 is a nuclear protein that is involved in tumor growth and overexpressed in multiple malignancies [38, 39]. Since WT1 is highly overexpressed in MPM, while its expression in lung adenocarcinoma is exclusively low, pathologists currently routinely use immunohistochemical (IHC) expression of WT1 for pathologic diagnosis of MPM [40]. Although WT1 is located in the nucleus, it can present on the cell surface with MHC molecules [38]. WT1 peptide vaccination for the treatment of MPM has yielded T-cell immune responses [41, 42]. Gene-modified WT1 TCR therapy is currently being studied in clinical trials (NCT02550535 and NCT01621724).

To develop personalized immunotherapeutic strategies for the treatment of MPM, we aimed to investigate the individual and coexpressions of MSLN, CA125, and WT1 in both epithelioid and non-epithelioid MPM. Heterogeneity of cancer-associated antigen expression in solid tumors allows for therapy resistance via antigen escape and is a known challenge for immunotherapies [43–45]. In anticipation of this challenge, we conducted a detailed analysis of the distribution of antigen-positive cells in each patient tumor for each cancer-associated antigen.

## RESULTS

### Clinicopathologic and demographic characteristics of patients

Among all 283 patients, 234 patients were diagnosed with epithelioid mesothelioma, 26 with biphasic mesothelioma, and 23 with sarcomatoid mesothelioma. Of the 234 patients with epithelioid mesothelioma, 39 patients were classified as having pleomorphic mesothelioma. The median age of patients with epithelioid mesothelioma and non-epithelioid mesothelioma (biphasic or sarcomatoid) were 63 years (range, 29-85 years) and 66 years (range, 41-79 years), respectively. The majority of patients were male (73% and 88% for epithelioid and non-epithelioid mesothelioma, respectively), positive for asbestos exposure (42% and 53%), smokers (58% and 57%), never received induction chemotherapy before surgery (70% and 80%), and were diagnosed with late-stage disease (stage III/IV, 68% and 84%) (Table 1).

**Table 1:** Malignant pleural mesothelioma patient characteristics

Variables	Epithelioid n = 234 (%)		Non-epithelioid* n = 49 (%)	
**Age (median, range)**	**63 (29–85)**		**66 (41–79)**	
**Gender**				
Female	64	(27.4)	6	(12.2)
Male	170	(72.6)	43	(87.8)
**Asbestos exposure**				
(+)	97	(41.5)	26	(53.1)
(-)	70	(29.9)	7	(14.3)
Unknown	67	(28.6)	16	(32.7)
**Smoking history**				
(+)	136	(58.1)	28	(57.1)
(-)	46	(19.7)	8	(16.3)
Unknown	52	(22.2)	13	(26.5)
**Induction chemotherapy**				
(+)	65	(27.8)	9	(18.4)
(-)	164	(70.1)	39	(79.6)
Unknown	5	(2.1)	1	(2.0)
**Stage**				
I, II	76	(32.5)	8	(16.3)
III, IV	158	(67.5)	41	(83.7)

* Biphasic or sarcomatoid subtypes

SD, standard deviation

### Intensity and distribution of antigen expression

Evaluation of MSLN, CA125, and WT1 were completed for 230 (98%), 226 (97%), and 226 (97%) cases of epithelioid MPM, respectively. For non-epithelioid MPM patients, evaluation of all 3 antigens was completed for 49 (100%) cases. Among epithelioid MPM cases, MSLN expression was positive in 93%, CA125 expression was positive in 75%, and WT1 was positive in 97% of cases. Distribution ≥50% of MSLN-, CA125-, and WT1-positive tumor cells were observed in 84%, 20%, and 72% of epithelioid MPM cases, respectively. Antigen expression intensity was: (1) strong in 35% and moderate in 34% of cases for MSLN expression; (2) strong in 9% and moderate in 27% of cases for CA125 expression; and (3) strong in 31% and moderate in 43% of cases for WT1 expression (Table 2). For non-epithelioid MPM cases, MSLN expression was positive in 57%, CA125 expression was positive in 33%, and WT1 expression was positive in 98% of cases. Distribution ≥50% of MSLN-, CA125-, and WT1-positive tumor cells were observed in 25%, 6%, and 45% of non-epithelioid MPM cases, respectively. Antigen expression intensity was: (1) strong in 8% and moderate in 16% of cases for MSLN expression; (2) strong in 0% and moderate in 4% of cases for CA125 expression; and (3) strong in 22% and moderate in 37% of cases for WT1 expression (Table 2).

**Table 2:** Distribution and intensity of cancer antigen expression in epithelioid and non-epithelioid malignant pleural mesothelioma

	Epithelioid						Non-epithelioid*					
	MSLN n = 230 (%)		CA125 n = 226 (%)		WT1 n = 226 (%)		MSLN n = 49 (%)		CA125 n = 49 (%)		WT1 n = 49 (%)	
**Expression**												
**Positive**	215	(93.5)	169	(74.8)	220	(97.3)	28	(57.1)	16	(32.7)	48	(98.0)
**Negative**	15	(6.5)	57	(25.2)	6	(2.7)	21	(42.9)	33	(67.3)	1	(2.0)
**Distribution**												
**≥50%**	193	(83.9)	46	(20.4)	162	(71.7)	12	(24.5)	3	(6.1)	22	(44.9)
**<50%, >0%**	22	(9.6)	123	(54.4)	58	(25.6)	16	(32.7)	13	(26.6)	26	(53.1)
**Intensity**												
**Strong**	81	(35.2)	20	(8.8)	71	(31.4)	4	(8.2)	0	(0.0)	11	(22.5)
**Moderate**	77	(33.5)	62	(27.4)	96	(42.5)	8	(16.3)	2	(4.1)	18	(36.7)
**Weak**	57	(24.8)	87	(38.5)	53	(23.5)	16	(32.7)	14	(28.6)	19	(38.8)

*Biphasic or sarcomatoid subtypes

CA125, cancer antigen 125; MSLN, mesothelin; WT1, Wilms tumor 1

### Coexpression of MSLN, CA125, and WT1

Concurrent evaluation of 3 antigens was completed for 220 (94%) epithelioid MPM cases and 49 (100%) non-epithelioid MPM cases. Triple antigen coexpression was demonstrated in 72% of epithelioid MPM cases and double antigen coexpression was demonstrated in 23% of epithelioid MPM cases. Triple and double antigen coexpressions were demonstrated in 29% and 33% of non-epithelioid MPMs, respectively. Only 1% of epithelioid and 2% of non-epithelioid MPM cases demonstrated complete absence of expression of any of the three antigens (Table 3).

**Table 3:** Coexpression of three cancer-associated antigens in epithelioid and non-epithelioid malignant pleural mesothelioma

	Epithelioid n = 220 (%)		Non-epithelioid* n = 49 (%)	
**Either antigen-positive**	218	(99.1)	48	(98.0)
**Triple-positive**	158	(71.8)	14	(28.6)
**Double-positive**	51	(23.2)	16	(32.7)
MSLN/CA125	3	(1.4)	0	(0.0)
MSLN/WT1	44	(20.0)	14	(28.6)
CA125/WT1	4	(1.8)	2	(4.1)
**Single-positive**	9	(4.1)	18	(36.7)
MSLN	1	(0.5)	0	(0.0)
CA125	0	(0.0)	0	(0.0)
WT1	8	(3.6)	18	(36.7)
**Triple-negative**	2	(0.9)	1	(2.0)

*Biphasic or sarcomatoid subtypes

CA125, cancer antigen 125; MSLN, mesothelin; WT1, Wilms tumor 1

### Coexpression of MSLN and CA125

Expression of either MSLN or CA125 was demonstrated in 96% of epithelioid and 57% of non-epithelioid MPM cases. MSLN/CA125 coexpression was demonstrated in 73% of epithelioid and 29% of non-epithelioid MPM cases. Single antigen expression of MSLN was exhibited in 21% of epithelioid and 14% of non-epithelioid MPM cases, whereas single antigen expression of CA125 was exhibited in only 2% of epithelioid and 4% of non-epithelioid MPM cases (Table 4).

**Table 4:** Coexpression of MSLN and CA125 in epithelioid and non-epithelioid malignant pleural mesothelioma

	Epithelioid n = 220 (%)		Non-epithelioid* n = 49 (%)	
**Both MSLN- and CA125-positive**	161	(73.2)	14	(28.6)
**Only MSLN-positive**	45	(20.5)	14	(28.6)
**Only CA125-positive**	4	(1.8)	2	(4.1)
**Both MSLN- and CA125-negative**	2	(0.9)	1	(2.0)

*Biphasic or sarcomatoid subtypes

CA125, cancer antigen 125; MSLN, mesothelin

### Distribution of antigen-positive cells by histologic subtypes

**Figure 1:**
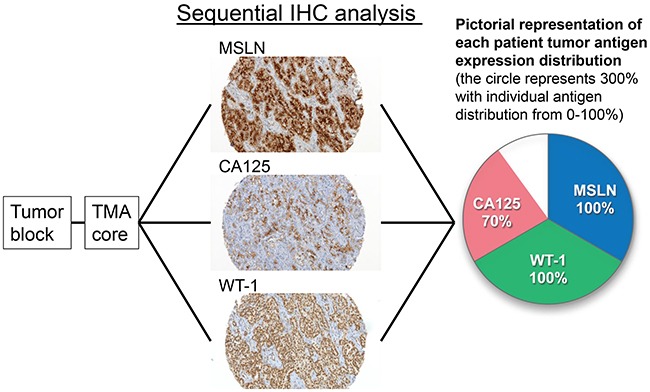
A representative case of sequential immunohistochemical analysis for distribution of antigen-positive cells Immunohistochemical staining for mesothelin (MSLN), cancer antigen 125 (CA125), and Wilms tumor 1 (WT1) were performed using sequential sections of tissue microarray blocks. Distribution of antigen-positive cells among tumor areas in each core was evaluated. The distribution of MSLN was 100%, CA125 was 70%, and WT1 was 100%. These values are shown in a pie graph for each patient.

**Figure 2:**
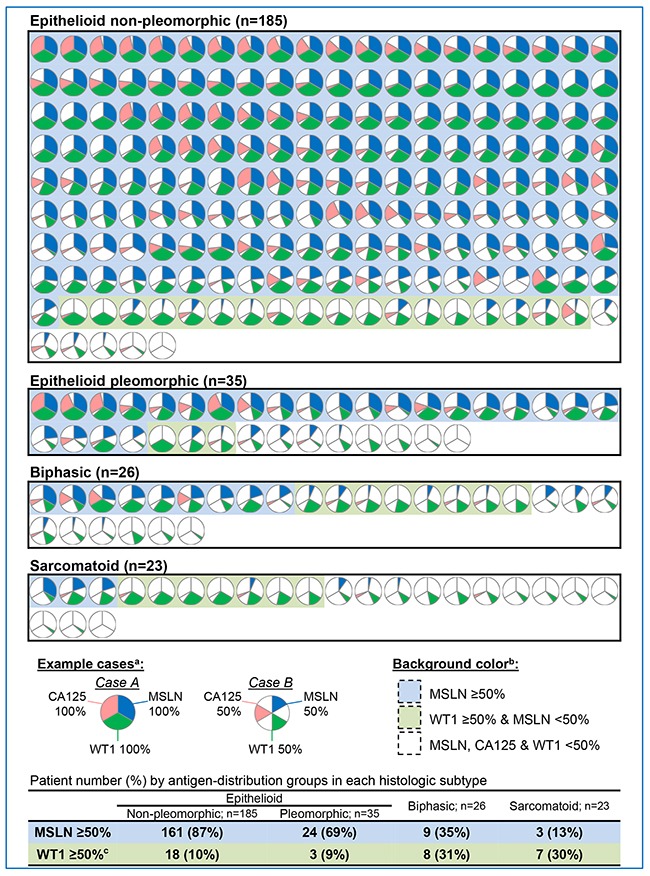
Antigen distribution pie graphs for each patient by histologic subtype Distribution of antigen-positive cells in a tumor area for mesothelin (MSLN), cancer antigen 125 (CA125), and Wilms tumor 1 (WT1) were evaluated and shown in a pie graph for each patient. The blue background represents cases with ≥50% MSLN distribution and the green background represents cases with <50% MSLN distribution and ≥50% of either CA125 or WT1 distribution. Cases without any antigen distribution ≥50% have no colored background. The tumors with ≥50% WT1distribution and <50% MSLN distribution. The 269 pie graphs were divided into four groups—non-pleomorphic epithelioid, pleomorphic epithelioid, biphasic, and sarcomatoid—and sorted by MSLN distribution for tumors with MSLN distribution ≥50%, followed by WT1 distribution in tumors with MSLN distribution <50%. In non-pleomorphic epithelioid MPM cases (n = 185), 87% had ≥50% MSLN distribution and 97% had ≥50% distribution of any single antigen. In pleomorphic epithelioid MPM cases (n = 35), 69% had ≥50% MSLN distribution and 77% had ≥50% distribution of any single antigen. In biphasic MPM cases (n = 26), 35% had ≥50% MSLN distribution and 65% had ≥50% distribution of any single antigen. In sarcomatoid MPM cases (n = 23), 13% had ≥50% MSLN distribution and 43% had ≥50% distribution of any single antigen.

The comprehensive data analysis of the distribution of antigen positivity for each individual patient is pictographically represented in Figure 1. Figure 1 is a representative case of three IHC sections. One section demonstrates the mean distribution of the antigen for the patient and the resultant patient pie graph. The distribution of antigen-positive cells for each of the three antigens in each patient is represented in Figure 2. Each pie graph represents the calculated antigen distributions of a single patient.

The 269 pie graphs were categorized into 4 groups—non-pleomorphic epithelioid, pleomorphic epithelioid, biphasic, and sarcomatoid. Given that our objective was to assess double and triple antigen coexpressions from the perspective of MSLN as the ideal antigen, we first sorted patients with MSLN-predominant tumors who were well-suited for MSLN-targeted therapy (i.e., MSLN distribution ≥50%). Subsequently, patients with MSLN distribution <50% were sorted by WT1 distribution. The blue background represents patients whose tumors had ≥50% MSLN distribution and the green background represents patients whose tumors had both <50% MSLN distribution and ≥50% WT1 distribution. Patients whose tumors had no antigen with ≥50% distribution have no background color associated with them.

For non-pleomorphic epithelioid MPM cases (n = 185), 161 (87%) patients had ≥50% MSLN distribution and 18 (10%) patients had ≥50% WT1 distribution without ≥50% MSLN distribution. For pleomorphic epithelioid MPM cases (n = 35), 24 (69%) patients had ≥50% MSLN distribution and 3 (9%) patients had ≥50% WT1 distribution without ≥50% MSLN distribution. For biphasic MPM cases (n = 26), 9 (35%) patients had ≥50% MSLN distribution and 8 (31%) patients had ≥50% WT1 distribution without ≥50% MSLN distribution. For sarcomatoid MPM cases (n = 23), 3 (13%) patients had ≥50% MSLN distribution and 7 (30%) patients had ≥50% WT1 distribution without ≥50% MSLN distribution. For all patients (n = 269), 197 (73%) patients had ≥50% MSLN distribution and 36 (13%) patients had ≥50% WT1 distribution without ≥50% MSLN distribution.

## DISCUSSION

In our study, we demonstrated that >98% of epithelioid and non-epithelioid MPM cases had positive expression of at least one of the three cancer-associated antigens. Positive expression included strong antigen expression, high frequency of double and triple antigen expressions, and high distribution of antigen-positive tumor cells. These findings, combined with the survival benefits shown in MPM patients with MSLN-, CA125-, and WT1-specific immune responses, provide the rationale for the development of targeted therapies. Additionally, the expression of these three antigens on normal tissue is very low [23, 34, 41] and this should be taken into consideration when developing new targeted therapies. Furthermore, published evidence has shown that antigen-specific spontaneous immune response are beneficial for different solid malignancies—MSLN in pancreatic cancer [32, 33], CA125 in ovarian cancer [34], and WT1 in mesothelioma [41]—and has driven us to explore the expression of these antigens on MPM. A growing number of immunotherapeutic investigations on the serial monitoring of these antigen-specific immune responses can further advance our understanding of immune responses and interactions for patients with MPM who have undergone treatment. The implications of intensity, distribution, and immune recognition of individual antigens should be kept in context when interpreting the antigen-specific immune responses.

Previous studies have reported that non-epithelioid subtypes were associated with worse prognosis compared with the epithelioid subtype in patients with MPM who were with surgery [46]. Additionally, we have reported that epithelioid MPM with pleomorphic features was associated with aggressive behavior and a similar survival rate to non-epithelioid MPM [47]. In our study, we investigated the individual and correlative expressions of three cancer-associated antigens, as well as their individual distribution in the tumor area, for each pathologic subtype. Interestingly, we found that these pathologic subtypes (non-pleomorphic epithelioid, pleomorphic epithelioid, biphasic, and sarcomatoid) had distinct antigen expression profiles, which are demonstrated by the proportion of patients with ≥50% distribution of each antigen. In non-pleomorphic epithelioid MPM, which is the most common MPM subtype, the majority of patients (87%) had ≥50% MSLN-positive cell distribution. Patients with pleomorphic epithelioid MPM also had a high frequency (69%) of ≥50% MSLN-positive cell distribution. Although the frequency of patients with ≥50% MSLN-positive cell distribution in non-epithelioid subtypes (biphasic, 35%; sarcomatoid, 13%) was lower than those in epithelioid subtypes, nearly one-third of these subtypes (biphasic, 31%; sarcomatoid, 30%) had ≥50% WT1-positive cell distribution. This suggests that, in those patients with ≥50% WT1-positive cell distribution without ≥50% MSLN-positive cell distribution, WT1-targeted therapy may be an alternative treatment option to MSLN-targeted therapy.

The pathologic and prognostic role of CA125 in MPM has not been elucidated. Previous studies have suggested that there is an interaction between MSLN and CA125 that may affect solid tumor metastatic potential [28–31]. There is an ongoing, early-phase clinical trial that has yielded promising results with blocking the MSLN/CA125 interaction using a chimeric MSLN-targeted antibody in combination with platinum-based chemotherapy [35–37]. In our study, CA125 overexpression was almost exclusively observed in MSLN-positive MPM. Additionally, more than two-thirds of epithelioid and one-third of non-epithelioid MPMs had MSLN/CA125 coexpression. These findings further support our rationale to target MSLN and suggest the potential for development of a MSLN/CA125 dual-targeted therapy that augments the effect of MSLN-targeted therapy.

One limitation of our present study is the use of a tissue microarray (TMA) that may not identify tumors that are focally positive on whole-tissue block staining. Despite this, we selected six TMA cores from six different areas in each tumor. We think that the frequency of each antigen overexpression and the distribution of antigen-positive cells in each patient will not be significantly changed if a whole-tissue block were used to confirm these results.

In conclusion, more than two-thirds of MPM patients have ≥50% distribution of MSLN-positive cells within whole tumor cells and, among the remaining one-third, half have ≥50% distribution of WT1-positive cells. CA125/MSLN coexpression was observed in more than two-thirds of epithelioid and one-third of non-epithelioid MPM patients. These results provide the rationale for developing personalized immunotherapeutic strategies for MPM patients that target these three cancer-associated antigens.

## MATERIALS AND METHODS

### Patients

The current retrospective study was approved by the Institutional Review Board (WA-0436-10) of Memorial Sloan Kettering Cancer Center (MSK). We reviewed all 620 patients who were diagnosed with MPM at MSK between 1989 and 2010. From this cohort, we reviewed 395 MPM cases with available hematoxylin and eosin (H&E)-stained slides. All slides were re-reviewed by two pathologists; this yielded 301 epithelioid, 59 biphasic, and 35 sarcomatoid MPM cases. Of these, 283 had tumor blocks available for construction of TMAs. Clinical data were collected from the prospectively maintained MPM database.

### Histologic evaluation

Histologic evaluation was performed using an Olympus BX51 microscope (Olympus, Tokyo, Japan) with a standard 22-mm diameter eyepiece. All tumors were classified as either epithelioid, sarcomatoid, or biphasic according to the 2015 World Health Organization classification [48]. For epithelioid mesothelioma, when cytologic pleomorphisms accounted for ≥10% of the tumor they were classified as a pleomorphic subtype [47].

### Tissue microarray

Formalin-fixed, paraffin-embedded tumor blocks were used for construction of TMAs. For epithelioid tumors, six to nine representative tumor areas were marked on H&E-stained slides. For biphasic tumors, six tumor areas were selected from a predominantly morphologic sarcomatoid lesion. Cylindrical 0.6 mm tissue cores were arrayed from the marked areas of corresponding paraffin blocks onto a recipient block using an automated tissue arrayer (ATA-27; Beecher Instruments, Sun Prairie, WI); this resulted in five TMA blocks.

### Sequential immunohistochemical analysis

Sequential paraffin 4 μm-thick sections were cut from the TMA blocks and deparaffinized. Sections were stained using a Ventana Discovery XT automated immunohistochemical stainer (Ventana, Tucson, AZ) for MSLN (5B2, MAb, Vector; diluted at 1:50) [21, 23], CA125 (OC125, MAb, Ventana; pre-diluted) [49], and WT1 (C19, PAb, Santa Cruz; diluted at 1:2000) [50] for IHC analysis. Cell conditioning solution (CC1) standard was used for heat-induced epitope retrieval for 60 min. Then, the slides were incubated by the biotinylated a-mouse secondary for MSLN and CA125 (Vector, diluted at 1:200), and a-rabbit secondary for WT1 (Vector, diluted at 1:200) for 60 min. The Ventana DAB MAP was used for visualization.

CA125 and MSLN expressions were mainly observed in the membrane of tumor cells and WT1 expression was observed in the nucleus. We evaluated the overexpression of these antigens by intensity of antigen expression and distribution of positive cells. The intensity of antigen expression for each core was determined by the pathologist as follows: 0 for no expression; 1 for weak; 2 for moderate; and 3 for strong. The intensity scores of six cores for each patient were averaged and rounded up to the next integer to obtain a mean intensity grade for each patient: 0 for no expression; 1 for weak; 2 for moderate; and 3 for strong. The distribution of antigen-expressing positive cells was determined as a percentage of the total number of cells, which is estimated in 10% increments. The antigen distribution of six cores for each patient was calculated and rounded up to the next 10% increment (i.e., a mean distribution score of 1-10% was assigned a distribution of “10%”).
